# Food Banks as a “Treasure Trove”: Users’ Experiences of a Western Australian Food Relief Organization

**DOI:** 10.3390/ijerph21081079

**Published:** 2024-08-16

**Authors:** Ned Marshall, Carolyn Bendotti, Jessica Charlesworth, Barbara Mullan, Chloe Maxwell-Smith

**Affiliations:** Behavioural Science & Health Group, School of Population Health, Curtin University, Bentley, WA 6102, Australia; ned.marshall@postgrad.curtin.edu.au (N.M.); carolynbendotti66@gmail.com (C.B.); barbara.mullan@curtin.edu.au (B.M.)

**Keywords:** food security, food banks, food relief, discretionary foods, food preferences

## Abstract

Food banks are providing crucial relief as food insecurity increases worldwide. While these services are essential for vulnerable populations, there is variability in foods available and users may experience poor nutritional quality, and an overabundance of discretionary foods, contributing to public health risks including overnutrition and obesity. Understanding how customers perceive food availability, variety, and quality is important to inform relief services and health interventions. This study reports the findings of a convergent parallel mixed-methods investigation of user experiences and perceptions of food availability, variety, and quality at a major food bank in Western Australia. Food bank customers (N = 207) at a food bank branch and mobile van locations completed a survey, with an option to complete a subsequent semi-structured interview (*n* = 15). Approximately 80% of the survey sample had low (48%) or very low (30%) food security, half of the sample had been using the food bank for longer than 6 months, and 77% reported the food bank as their first choice for food. Three-quarters (77%) reported financial barriers to a balanced diet in the past twelve months and described how limited availability and variety complicated shopping. Interviewees explained complex perceptions of these issues, including favouring healthy food while considering discretionary food as a “luxury” that enhanced their quality of life. Our findings suggest that food bank users experience barriers to maintaining a balanced diet, encounter variable supplies of healthy and nutritious foods, and have concerns about the impacts of frequent discretionary food consumption. These findings have implications for public health promotion.

## 1. Introduction

Food security is a key determinant of health, existing when individuals and communities have physical, social, and economic access to healthy food [1]. Food insecurity occurs when the physical or mental well-being of an individual or community is at risk because of poor access, availability, utilisation, or stability of nutritious food [1]. Worldwide, approximately 2.4 billion people are moderately or severely food insecure [2]. A range of factors have recently contributed to worsening food insecurity internationally, including political conflict, climate crises, and COVID-19 [3].

There is a paradox in Australia’s food insecurity. Australia is a high-income country that is not food insecure according to the Department of Agriculture, Fisheries, and Forestry Australia, yet food relief organisations are struggling to meet significant increases in demand [4,5]. Approximately 3.7 million Australian households experienced food insecurity in the 2022–2023 period, an increase of 1.7 million from the previous year [6,7]. Food insecurity is associated with health risks including undernourishment, wasting, stunting, underweight, or overweight [8,9]. These health risks can be present at food banks where nutrient-poor, high-sugar, and high-fat food donations are often readily available and low in cost [10,11]. Energy-dense, nutrient-poor foods are often termed ‘discretionary foods’ and include confectionary, crisps, and sugar-sweetened beverages [12]. Nutritionally poor diets are a major preventable lever for non-communicable diseases [13], and have a significant economic burden [14]. Discretionary and ultra-processed foods are also unsustainable, with a negative impact on public health and the environment [15].

Food banks and food pantries are imperative to provide food relief [16]. Food banks often function as a warehouse, receiving donated food and distributing it to smaller relief organisations, while food pantries resemble a small food market but offer subsidised or free food. We use the term ‘food bank’ from here on, as the organisation we recruited from is named ‘Foodbank WA’, operates both a warehouse and pantry, and was referred to by participants as ‘Foodbank’ or ‘the food bank’. Food banks offer more choice than some food relief models, including pre-packaged hampers, pre-made meals, and referral services. Many Australian food relief organisations have struggled to meet customer’s needs, due in part to, limited staffing and funding, inconsistent adherence to nutritional policies, and the prevalence of unhealthy food donations [17]. Foodbank Australia is one of Australia’s largest food relief services, and demand in Western Australia, at Foodbank WA, grew from 200 people per day before the pandemic to 700 per day at peak times during the pandemic [6]. Despite users’ reliance on food banks, the effectiveness of the food bank model as a solution to food insecurity has been questioned, given the difficulty with meeting demand [18,19]. Research has suggested limited availability, variety, and quality of food donations are reported by users as limitations of food banks [20,21,22].

The reported issues at food banks warrant research to understand user characteristics and to explore user perceptions of donated food and food relief services. This can elucidate whether needs are being met, inform health promotion efforts, and improve food bank services. The current study employed a convergent-parallel mixed methods design with a survey and semi-structured interviews to answer the following research questions:

RQ 1. What are the characteristics of users of a Western Australian food bank?
RQ 2. What are users’ experiences of availability, quality, and variety of foods at the food bank?

## 2. Materials and Methods

This study was part of a broader research initiative between Curtin University and Foodbank WA to identify the demographics of food bank users, and experiences of food relief and food insecurity. Ethics approval was obtained from the Curtin University Human Research Ethics Committee (HRE2023-0178).

### 2.1. Participants

To be eligible for inclusion in the study, participants were adult customers who had accessed Foodbank WA. Participants were recruited face-to-face at the Foodbank warehouse and at 9 mobile van locations across metropolitan Perth, Western Australia. Purposive sampling was employed for this research since having accessed a food bank was core to the study aim. Demographics can be seen in Table 1. Researchers handed out flyers with QR codes, paper surveys, and iPads with the Qualtrics survey loaded for offline use. After removing completely missing cases, survey participants (*N* = 183) were from 66 different postcode locations.

Of the 183 participants who completed the survey, 64 participants expressed interest in an interview and were emailed a participant information sheet and consent form, from whom 19 responded. Information power informed the sample size for interviewees (n = 15) and interview data collection ceased when information power was met [23]. Interviews took place from June to August 2023 and the average interview time was 30 min (range: 14–47 min). Interview demographics were similar to overall survey demographics; the majority of interview participants identified as women (73%) and had very low food security (60%). The average age of participants was 51 years old (range: 28 to 77 years).

### 2.2. Measures

#### 2.2.1. Survey

The survey was designed collaboratively with Foodbank WA staff and comprised 43 items. The survey included items to assess demographic characteristics, food security, and service satisfaction. Demographic items included age, gender identity, living situation, parental status, level of educational attainment, gross annual household income, main source of income, and current employment status.

Food security was assessed using the short-form 6-item Household Food Security Survey Module (HFSSM) [24]. The 6-item Likert-scale questionnaire has previously accurately identified 97.7% of families living with food insecurity [25] and has been used in recent Australian research [26]. The descriptive classifications indicate whether people have ‘high or marginal food security’ (raw score 0–1), ‘low food security’ (raw score 2–4), or ‘very low food security’ (raw score 5–6). An example question is “in the last 12 months, did you or other adults in your household ever cut the size of your meals or skip meals because there wasn’t enough money for food?” For this study, high and marginal food security were grouped and considered ‘food secure’, while low and very low food security were grouped and categorised as ‘food insecure’, as described by the 6-item HFSSM instructions and as has been reported in other Australian research using variations of the HFSSM [19,27]. Service satisfaction items were developed from consultation with Foodbank WA staff and included items measuring satisfaction with availability, quality, and variety.

#### 2.2.2. Interviews

The semi-structured interview schedule opened with the interviewer describing the project and reiterating the interview procedure. Interviews explored experiences of food bank use, food insecurity, food availability, and accessibility. Example questions include ‘How would you describe your usual experience at Foodbank?’ and ‘Can you talk about your experiences running low on food or worrying about running out of food?’. Care was taken to sensitise interview questions and reduce risk of distress.

### 2.3. Procedure

Researchers distributed the survey at Foodbank WA headquarters and metropolitan locations of the Foodbank mobile van service between June and July 2023. Interested users provided informed consent prior to completing the 10 min digital or paper survey. Participants had the opportunity to express interest in being interviewed at the end of the survey and were subsequently contacted by email by a researcher. Interviews were conducted via phone call or Microsoft Teams and recorded, transcribed, de-identified, and uploaded to NVivo. Interviews took place between June and August 2023. Participants received a AUD 5 food bank voucher for survey participation and a AUD 15 voucher for interview participation.

### 2.4. Analysis

Analysis of quantitative data involved descriptive statistics, a multiple regression (Table 2, Table 3 and Table 4), Mann–Whitney U tests (Table 5), and partial correlations (Table 6) to understand the characteristics and factors associated with food insecurity and food bank use in this population. A regression was used to estimate the proportion of variance in household food security that can be accounted for by age, gender, income, and level of educational attainment.

For the multiple regression, gender and level of educational attainment were not assessed for normality, due to the nature of these categorical variables, but normality for age was reasonable. Income was positively skewed, as expected for this sample. Probability plots and standardized residual scatterplots showed assumptions of normality, linearity, and homoscedasticity were met, and multivariate outliers were considered non-influential (i.e., No Cook’s Distance values exceeded 1, and all tolerance and VIF were close to 1).

Partial correlations measured the relationship between customer satisfaction with quality, quantity, and variety of donated food, while controlling for potential confounders including age, gender, education, and income. Reflexive thematic analysis was used to analyse the qualitative data (Table 7) [28].

## 3. Results

About half of the participants (51%) used the Perth warehouse headquarters, 40% used a mobile van location, and 9% reported mainly using a food bank outside of the metro area. Of the respondents, 22% had high or marginal food security, 48% had low food security, and 30% had very low food security. Three-quarters of participants identified as women (74%) and 23% as men, and 3% self-identified as a non-binary gender. The sample had an average age of 40 and a standard deviation of 14. More than half of the participants were born in Australia (64%), followed by the United Kingdom (14%) and New Zealand (4%). Ten percent of respondents identified as Aboriginal or Torres Strait Islander.

Most customers (74%) listed government benefits as their primary source of income, and 83% reported receiving less than AUD 50,000 annual income before tax. Seventeen percent were receiving disability support payments. Half (50%) were supporting children, of whom 34% were supporting two or more children. Thirty percent were single parents. Thirty-four percent of participants were renting, and 18% were homeowners. Slightly more than half of respondents (53%) reported having a tertiary qualification.

Seventy-one percent reported they had not used the food bank before the COVID-19 pandemic. Half of the respondents (49%) had been using the food bank for 6 months or longer. Of this, 18% had been using the food bank for 6 to 12 months, and 31% reported using the food bank for more than a year. People generally used the service weekly (43%) or fortnightly (28%). About a quarter (26%) had required emergency (free) food from Foodbank and 43% had received emergency food relief from another service.

The regression model explained a moderate amount of variability in household food security (13%). In combination, age, gender, income, and education accounted for a significant 13% of the variability in household food security, $R^{2}$ = 0.13, adjusted $R^{2}$ = 0.106, *F* (4,144) = 5.366, *p* = 0.001. A Mann–Whitney U test showed no significant difference in food security scores between parents (Mean Rank = 80.30, *n* = 81) and non-parents (Mean Rank = 68.69, *n* = 68), *U* = 2325.00, *z* = −1.679, *p* = 0.093.

### 3.1. Food Banks as a Treasure Trove: Availability and Variety

There were diverse experiences of availability and variety at the food bank. While some participants experienced wide availability and high variety, others experienced narrow availability of foods and low variety. This contrast supports the idea that overall variety is an important element for users’ food preferences. There was also nuance in how people considered food as healthy, useful, and quality, and what foods were considered as “luxury”.

More than two-thirds (77%) reported that the food bank was their first choice for grocery shopping. Many participants expressed demand for items beyond food, including hygiene and cleaning products. Most respondents (81%) were satisfied with the value for money. Of the survey items on satisfaction (quality, quantity, variety, wait time), participants were least satisfied with the variety of foods available, with 39% reporting some degree of dissatisfaction. Some customers experienced high availability and variety, referring to the food bank as “a treasure trove” (P15/W/high FS) and experienced “quite a different array” (P6/W/low FS). Despite describing the food bank as a “treasure trove”, participant 15 suggested this was not consistent and depended on what had been donated: “They only get what is supplied to them and it’s variable, like very variable” (P15/W/high FS). Perceptions differed on the extent of this variability and the influence it had on users’ shopping experience and diet management.

There were significant positive correlations between satisfaction items, while controlling for confounding variables of user characteristics of age, gender, education, and income. Quantity and variety (*r_s_* = 0.645, *p* < 0.001, two-tailed, *df* = 142), quantity and quality (*r_s_* = 0.612, *p* < 0.001, two-tailed, *df* = 142), and quality and variety were highly positively correlated (*r_s_* = 0.677, *p* < 0.001, two-tailed, *df* = 142). This indicates that experiences of the availability (quantity), quality, and variety of donated food are related to customer satisfaction at the food bank and are not influenced by these user characteristics.

When variety and availability were high, this provided choice and satisfaction with the food bank. Participants suggested this improved their experience and helped them source a more balanced diet: “I feel actually more spoilt for choice now than if I wasn’t going to Foodbank. Then my selection would be even more limited because I would have to pick the cheapest options” (P15/W/high FS). This suggests availability and variety are supportive factors of food bank use. This sentiment was reflected in another participant’s positive experience with availability: “…I like the range. Everything I need, I get there…” (P10/W/very low FS). The positive impact of availability and variety empowered the agency of users, alluding to feelings of independence and dignity:

“You feel like a human. You feel like you have the power to choose what you want to feed your children. You’re given the power, the opportunity, the respect to do what you want to cook. So, it’s something that I have never experienced so I’m grateful” (P10/W/very low FS).

The consequences of low availability and variety were two-fold. Firstly, it seemed to make sourcing a balanced diet complicated for some: “… it’s an eclectic mix that… it sort of does ease the burden a bit, but it doesn’t really meet your food needs” (P4/W/low FS). As previously mentioned, this variability was attributed to donations, and food bank processes: “… you really can’t plan, meal plan. You can’t do anything like that because it just depends on what they’ve got in at the time” (P2/W/very low FS). Participants reported seeing available products in the warehouse that they wanted to purchase, but these were stacked high on shelves for distribution at a later date.

Secondly, variability meant planning was more time-consuming for participants, who had to shop at a variety of other places for meal provision: “going to the food bank is a more time resource expense…” (P1/W/very low FS). While standard supermarkets provide a reasonably consistent supply of the same foods, food banks may not always provide this same assurance. This meant shopping at the food bank required being “savvy”, “thinking outside the box”, or being “the mother of invention”. Examples included shopping between multiple supermarkets for specials and discounts, meal planning, sharing with friends or family, and “stretching” money to cover food costs. Further indicating food bank user interest in utility and resourcefulness, 35% of survey participants said they would be interested in cooking classes and recipes. At the extreme, the time cost of meal-planning for a family impacted the ability to work: “People say ‘oh, get out and get a job’ or ‘do part time work’ but you know, it’s a difficult sort of thing because you’re spending so much time putting food on the table from different sources” (P4/W/low FS). Another participant who did multiple shops considered himself “lucky” to have the time to do this (P5/M/very low FS).

The concept of time cost was supported by reported wait times at the food bank and a perceived increase in demand. Survey respondents listed an average wait time of 18 min with a standard deviation of 14 min. Wait time had lower satisfaction (69%) than other items. Long wait times were noted in open-text responses about dissatisfaction and attributed to increasing demand:

“The numbers of people visiting Foodbank has increased significantly, which is having an impact on check out times—there are only two tills available. Staff do a fantastic job and are working as hard as they can, there just aren’t enough of them” (P184/W/High FS).

The thread between these experiences is variability. People often recounted experiencing high and low availability, or luxury and poor quality, at different visits. High availability and variety provided agency to customers, and was supportive of nutrition needs, dietary needs, meal planning, and access to the items needed for a balanced diet.

### 3.2. Discretionary Food as a Luxury

Experiences of food donation quality seemed to be informed by perceptions of healthiness and luxury. Participant 12 suggested “I do love the variety of things that are there” but also suggested the quality was not empowering: “… using food bank is surviving. It’s not thriving.” (P12/W/low FS). Accordingly, 77% of survey respondents reported they could not afford to eat balanced meals in the past 12 months. The impact on quality of life and health was often tied to the presence of unhealthy or discretionary foods: “They tend to have a lot of junk food there, but they don’t tend to have a lot of healthier-style foods” (P1/W/very low FS). While participants generally expressed a desire for healthy foods, for some, the availability of discretionary foods was a “luxury” and perceived as a treat: “…last time I went, I got some ice cream… That’s just a luxury we just don’t have…” (P4/W/low FS).

The perceived availability of discretionary food, or “junk food”, was sometimes seen as positive since it contributed to overall availability: “I mean we all need to have some junk food because it’s, you know, it’s fun and just because you’re poor shouldn’t mean that you don’t get to have any fun” (P12/W/low FS). For people who did not personally appreciate the availability of discretionary foods, they still saw benefit in this for families, and children who would more easily eat these foods: “I call it rubbish food. I wouldn’t eat it but, you know, it’s great for a family” (P9/W/very low FS). Participants experiencing low food security were cautious of wasting any food, and discretionary foods were seen to have a lower risk of food waste from children. Luxury was also referenced in relation to typically expensive options being available and affordable at the food bank: “You can get some of the luxury items that you just can’t afford at the supermarket” (P14/M/very low FS).

Participants generally expressed a desire and preference for healthier foods. When this was not the case, it was because healthier foods like fresh produce had a greater risk of quick expiry or wastage due to children not wanting to eat them.

### 3.3. Donation Quality and Safety

Perceptions of quality varied, especially for fresh produce. These were generally occasional encounters and not necessarily consistent experiences. Unsafe food was generally linked to food donors. Two main concerns resulted from experiences with poor quality food: fear of wastage and fear of contamination. At the extreme, some food, in particular fresh produce, was described as: “…on its last legs.” (P4/W/low FS). Some participants recounted purchasing expired food, or food close to expiry: “I did get a fresh produce bag last week. And a lot of that I couldn’t eat ‘cause it had gone off. So that was a little bit disappointing, but the quality of the food is usually really good” (P6/W/low FS). These experiences fuelled concerns about food wastage and increased the risk of inedible food: “I’ve found that a lot of it went to waste before you got to use it…” (P15/W/high FS). People who had negative experiences were able to return or exchange this at the food bank without hassle, although there was sometimes hesitance due to how they might be perceived: “… it’s all donated so you can’t sort of be picky…” (P9/W/very low FS). Participants discussed making concerted efforts to avoid waste, and if they encountered poor quality food or food close to expiry, they would give it away so that it wasn’t wasted: “… usually there’s pretty good range. Sometimes there isn’t though, and I, you know, I had to give some of it away” (P9/W/very low FS). One participant expanded by discussing the dilemma between hunger and risk of contamination:

“It’s really stressful because on the one hand, you sort of know you’ve gotta eat. And on the other side, you’ve got in the back of your mind, ‘is this actually good enough to eat or am I actually gonna poison the kids cause this is out of date?” (P4/W/low FS).

Despite overall satisfaction with the food bank, participants noted occasional experiences with expired or unsafe donated food, resulting in stress about their well-being, wastage, or hesitance to return or exchange expired food due to guilt or inconvenience.

## 4. Discussion

This study contributes to the body of evidence on experiences at food banks. Our findings concern the equity and health of food bank users, supporting the need for policy action from government, nutrition policy for food donations in Australia, and health promotion intervention for food literacy and food safety knowledge. Solutions should involve addressing the causes of food insecurity, as well as addressing the consequences [29].

Our results highlight inequality and inequity of food bank users, with a high representation of people receiving welfare payments, women, single parents, and older people. Women tend to be responsible for a disproportionate amount of food procurement and meal preparation in households [30]. This also concerns children, as 50% of participants reported supporting children, and food security measures have shown to be predictors of child food security [31]. The regression model showed that age, gender, education, and income predicted 13% of the variance in household food security, suggesting other variables influence household food security (Table 2). Government policy action, and support for health promotion through the food bank, can support food security and reduce these inequities [32]. Solutions include increasing welfare payments to meet an increasing cost of living [33]. Action could also involve a National Food Plan, a National Food Council, and a school meal program, all recommended by a recent parliamentary inquiry into Australia’s food security [34]. This plan would take a systems perspective and be overseen by the Council. Food policy groups (similar to the proposed National Food Council) have been shown to have a positive impact on food security and nutrition by increasing political accountability, and setting targets that can be evaluated [35,36]. Formally recognizing the right to food in Australian law could also protect these measures and support long-term food security [37,38].

Our findings highlight the poor dietary health of people experiencing food insecurity, with three-quarters (77%) of this sample struggling to afford a healthy diet. While the general population often have poor adherence to dietary guidelines [39], people experiencing food insecurity may be more at risk of poor diet quality due to dependence on donated food [21,26,40,41]. Customers’ food perceptions underscore the need for nutritional guidelines for donated food, alongside recent audits of the nutritional value and safety of donated foods [42,43]. Significant satisfaction between quantity, variety, and quality indicates these are related concepts for consumers, regardless of their age, gender, education, or income (Table 6). As well as nutritional and food safety guidelines, other potential policy solutions include the proposed Food Donation Tax Incentive, designed to address the poor availability of healthy foods at food relief organisations by incentivising healthier donations and addressing food waste [44].

While nutritional guidelines for donated food offer a solution to the cause of this issue [11], health promotion interventions can address the health consequences. Previous research has suggested that poor ‘food literacy’ (dietary health knowledge and skills) is related to food insecurity [45,46]. Food literacy includes concepts like food resource management, involving the optimization of procuring, storing, and preparing food to meet dietary needs with available resources while minimizing waste.

Behaviour change campaigns and education programs can improve well-being and food security. While food bank users can be limited by availability and cost [47,48], health promotion interventions like SWAP (Supporting Wellness at Pantries) have been shown to support healthy and nutritious food selection in the United States [49,50]. Other programs like the US ENFEP (Expanded Food and Nutrition Education Program) [51], OzHarvest’s NEST (Nutrition Education and Skills Training) [52], and Foodbank’s Healthy Food for All [53] have been shown to improve both food literacy and food security. In two years (2022–2023), Foodbank WA’s Healthy Food for All program ran 229 sessions with 748 participants. Our data support the expansion of food literacy programs like Healthy Food for All, as one-third (35%) of participants in our study said they would like to receive recipes and cooking classes. Consumer’s preference for healthy and safe food in this study shows further support, as an intention to avoid discretionary foods provides an opportunity to tailor more effective health promotion interventions [54]. Consumer concern about food safety in this study also supports the role of food safety and date label education. Knowledge of food safety is low among consumers in Australia, and this is an area for improvement [55]. Behaviour change campaigns involving education, habit formation, and risk perception can be effective in supporting consumer food safety knowledge [56,57,58]. Education about food safety can improve well-being, support public health, reduce food waste, improve sustainability, and have particular benefits for vulnerable populations [59,60]. Motivation can be a barrier to food literacy programs [61] but strategies like supporting self-efficacy [62] and setting outcome expectancies [63] can help address this. While food literacy programs and health interventions are beneficial, there is a risk of governments outsourcing responsibility to charities and not-for-profit organisations, rather than addressing root causes of food insecurity like income inequality [64,65,66], bringing into question what governments consider to be acceptable levels of food insecurity [67].

Lastly, our findings support a six-factor model of food security, which includes agency and sustainability along with access, availability, utility, and stability [68]. People valued high availability, and the choice model at the food bank empowered consumers’ shopping experiences. The value of agency is also supported by the nuance people expressed in their opinions about unhealthy options as ‘luxury’ and ‘fun’. As well as improving satisfaction, food bank models that incorporate choice can also have more of an impact on reducing food insecurity [69]. Choice is instrumental for people experiencing food insecurity. This reflects the concept of food sovereignty: the notion that individual agency is a prerequisite for food security [38]. Accordingly, an alternative concept to food security, ‘food justice’ has been supported by a growing movement to reinforce food access as a human right and to recognise that traditional relief efforts have been largely ineffective [70].

### Strengths and Limitations

Strengths of this research include a broad sampling technique that recruited across metropolitan Perth, with an exploration of a topical issue that harnesses the potential to empower people who may be disenfranchised by their food security status. The survey served both academic and practical interests across the research team and Foodbank WA. However, research may seek larger sample sizes and minimise survey length for practicality and translation. Exploration of these issues with other samples, such as volunteers, could offer perspectives on food availability and consumer behaviour to provide a unique, organisational lens. These endeavours could then inform public perceptions of food insecurity, knowledge about food relief organisations, and the level of support for intervention, guiding policy and public health solutions.

## 5. Conclusions

This research explored customer characteristics and experiences of a food relief organisation in Western Australia. Data suggest that while vulnerable populations using food banks have high satisfaction and can view them as a ‘treasure trove’, they still have concerns about managing a healthy and balanced diet, due to variable food donation type and quality. Users are aware of unhealthy and discretionary foods, sometimes choosing these options for their convenience and low risk of waste. Policies and programs at government, organizational, and consumer levels can reduce health inequities, while harnessing food bank user well-being as a public health interest.

## Figures and Tables

**Table 1:** Descriptive statistics.

Characteristics	*N*	%
Gender		
Woman	135	73.8
Man	42	23.0
Self-describe	5	2.7
Missing	1	0.5
Country of Birth		
Australia	107	63.7
United Kingdom	21	13.8
New Zealand	7	4.2
South Africa	4	2.4
Afghanistan	3	1.8
China	2	1.2
India	2	1.2
Philippines	2	1.2
Syria	2	1.2
USA	2	1.2
Zimbabwe	2	1.2
Other (<2)	7	4.2
Missing	22	2.7
Identify as Aboriginal or Torres Strait Islander		
Yes	19	10.4
No	154	84.2
Prefer not to say	8	4.4
Missing	2	1.1
Living situation		
Living alone	46	25.1
Not living alone	135	73.8
Missing	2	1.1
Current Accommodation		
Private Rental	62	33.9
Department of Communities Housing	50	27.3
Homeowner (mortgage)	32	17.5
Homeowner (no mortgage)	14	7.7
Emergency Accommodation/Hostel	4	2.2
Living on the Street	5	2.7
Prefer not to say	5	2.7
Other	8	4.4
Missing	3	1.6
Adults in household		
1	61	33.3
2	59	32.2
3	27	14.8
4 or more	32	17.5
Missing	4	2.2
Children in household		
0	85	46.4
1	29	15.8
2	29	15.8
3 or more	34	18.6
Missing	6	3.3
Single parent household		
Yes	55	30.1
No	43	23.5
No children at home	81	44.3
Missing	4	2.2
Combined gross annual household income before tax		
Negative income	7	4.1
No income	18	10.6
Less than AUD 10,000	10	10
AUD 10,000–30,000	68	40.0
AUD 30,000–50,000	38	22.4
AUD 50,000–70,000	6	3.5
AUD 70,000–90,000	3	1.8
AUD 90,000–110,000	1	0.6
AUD 110,000–130,000	1	0.6
AUD 130,000–150,000	1	0.6
More than AUD 150,000	2	1.2
Missing	37	17.9
Current employment status		
Full-time	9	4.9
Part-time	19	10.4
Casual	18	9.8
Government assistance	40	21.9
Government disability support	31	16.9
Home duties	29	15.8
Student	5	2.7
Retired	19	10.4
Prefer not to answer	6	4.9
Missing	4	2.2
Main source of income		
Government benefits	135	73.8
Wage or salary	20	10.9
Family, friend, or partner	12	6.6
No income	8	4.4
Missing	8	4.4

*N* = 183. Participants were on average 40.2 years old (SD = 13.6).

**Table 2:** Model summary of regression.

Model		Sum of Squares	df	Mean Square	F	Sig.
1	Regression	95.057	4	23.764	5.366	<0.001
	Residual	637.735	144	4.429		
	Total	732.792	148			

Dependent Variable: Household Food Security Survey Module; Predictors: (Constant), level of educational attainment, age, gender, combined gross annual household income.

**Table 3:** ANOVA.

Model	R	R Square	Adjusted R Square	Std. Error of the Estimate
1	0.360	0.130	0.106	2.10445

Predictors: (Constant), level of educational attainment, age, gender, combined gross annual household income; Dependent Variable: Household Food Security Survey Module.

**Table 4:** Coefficients.

Model		Unstandardized Coefficients		Standardized Coefficients	t	Sig.	Collinearity Statistics	
		B	Std. Error	Beta			Tolerance	VIF
1	(Constant)	8.056	1.154		6.980	<0.001		
	Age	−0.054	0.014	−0.316	−3.867	<0.001	0.908	1.101
	Gender	−0.177	0.364	−0.039	−0.487	0.627	0.925	1.081
	Combined gross annual household	−0.134	0.079	−0.138	−1.690	0.093	0.909	1.100
	Level of educational attainment	−0.392	0.186	−0.170	−2.102	0.037	0.926	1.080

Dependent Variable: Household Food Security Survey Module.

**Table 5:** Mann-Whitney U Test comparing food security of parents and non-parents.

Measure	Non-Parents (n = 68)	Parents (n = 81)	U	Z	*p*
Food security (Mean Rank)	68.69	80.30	3183.00	1.679	0.093

**Table 6:** Partial correlations between satisfaction with quality, quantity, variety, controlling for age, gender, education, and income.

		Satisfaction with Quality	Satisfaction with Quantity	Satisfaction with Variety
Satisfaction with quality	Correlation	1.000		
	Significance (2-tailed)			
	df	0		
Satisfaction with quantity	Correlation	0.612	1.000	
	Significance (2-tailed)	<0.001		
	df	142	0	
Satisfaction with variety	Correlation	0.677	0.645	1.000
	Significance (2-tailed)	<0.001	<0.001	
	df	142	142	0

Note: all partial correlations control for age, gender, education, and income.

**Table 7:** Qualitative data.

Theme	Example Quote 1.	Example Quote 2.	Example Quote 3.
**Food banks as a treasure trove: availability and variety:** This theme represents the nuance of customer experience at the food bank including high and low availability and variety, and the personal effects of these, including empowerment when availability and variety are high, and a time cost when availability are variety are low. **Empowerment (availability and variety high):** This theme represents situations where customers experienced high or abundant food sources at the food bank. **Time cost (availability and variety low):** This theme represents situations where customers experienced difficulty from low or limited food sources at the food bank.	“You’re given the power, the opportunity, the respect to do what you want to cook.”	“…I like the range. Everything I need, I get there…”	“I feel actually more spoilt for choice now than if I wasn’t going to Foodbank. Then my selection would be even more limited because I would have to pick the cheapest options”
	“going to the food bank is a more time resource expense…”	“People say ‘oh, get out and get a job’ or ‘do part time work’ but you know, it’s a difficult sort of thing because you’re spending so much time putting food on the table from different sources”	“… you really can’t plan, meal plan. You can’t do anything like that because it just depends on what they’ve got in at the time”
**Discretionary food as a luxury:** The central organising concept of this theme is the idea that people value discretionary food. This includes as a comfort, for the ease of catering to children’s wants, and reducing chance of wastage.	“…last time I went, I got some ice cream… That’s just a luxury we just don’t have…”	“I mean we all need to have some junk food because it’s, you know, it’s fun and just because you’re poor shouldn’t mean that you don’t get to have any fun”	“You can get some of the luxury items that you just can’t afford at the supermarket”
	“They tend to have a lot of junk food there, but they don’t tend to have a lot of healthier-style foods”	“I call it rubbish food. I wouldn’t eat it but, you know, it’s great for a family”	“I do love the variety of things that are there… using food bank is surviving. It’s not thriving.”
**Donation quality and safety:** This theme explores customer’s experiences of the nutritional quality of donated food, perceptions of food safety in donated products, and how they respond to these issues.	“I did get a fresh produce bag last week. And a lot of that I couldn’t eat ‘cause it had gone off. So that was a little bit disappointing, but the quality of the food is usually really good”	“I’ve found that a lot of it went to waste before you got to use it…”	“… it’s all donated so you can’t, sort of be picky…”
	“… usually there’s pretty good range. Sometimes there isn’t though, and I, you know, I had to give some of it away”	“It’s really stressful because on the one hand, you sort of know you’ve gotta eat. And on the other side, you’ve got in the back of your mind, ‘is this actually good enough to eat or am I actually gonna poison the kids cause this is out of date?”	“…on its last legs.”

## Data Availability

De-identified data are available upon request.
